# The Associations of Income, Education and Income Inequality and Subjective Well-Being among Elderly in Hong Kong—A Multilevel Analysis

**DOI:** 10.3390/ijerph17041271

**Published:** 2020-02-17

**Authors:** Eric TC Lai, Ruby Yu, Jean Woo

**Affiliations:** Jockey Club Institute of Ageing, Chinese University of Hong Kong, Hong Kong SAR, China; rubyyu@cuhk.edu.hk (R.Y.); jeanwoowong@cuhk.edu.hk (J.W.)

**Keywords:** subjective well-being, elderly, income inequality, Hong Kong

## Abstract

*Background:* Higher income and education and lower income inequality in a neighbourhood have been shown to be related to better mental health outcome in developed countries. However, it is not clear whether these factors would affect the subjective well-being of the elderly, especially in a setting with recent rapid economic development. *Methods:* This study was conducted in 80 community centres with a total of 7552 community-dwelling elderly (mean age 75.9 years (SD = 7.79), 79% female) in Hong Kong. Income at individual level was measured as perceived disposable income. Education level was also collected. At district level, income was measured by district median household income and education was measured as the proportion of the population with no formal schooling. Income inequality was quantified using Gini coefficients. Low subjective well-being was defined as any one or a combination of the following: not satisfied with life, no meaning of life and being unhappy (Likert scale ≤ 2). Multilevel logistic regression was used to assess the association of income, education and income inequality and low subjective well-being. *Results:* We found that 15.3% (95% confidence interval (CI): 14.5 to 16.1) of the elderly have low subjective well-being. Compared with elderly who reported a very adequate disposable income, those who reported a very inadequate disposable income are at increased risk of low subjective well-being (OR=5.08, 95%CI: 2.44 to 10.59). Compared with elderly with tertiary education, those with no formal schooling were at higher risk (OR=1.60, 95%CI 1.22 to 2.09). Income inequality was not related to subjective well-being. *Conclusions*: Elderly with inadequate disposable income and lower education level are more likely to suffer from low subjective well-being. At the neighbourhood level, income inequality was not related to subjective well-being. However, the relationships between neighbourhood income and education level and individuals’ subjective well-being are not clear.

## 1. Introduction

Subjective well-being has been gaining traction in the public policy research arena as researchers and policy-makers are striving to improve people’s well-being. A commission group initiated by the French government and chaired by Joseph Stiglitz advocated that when measuring social progress, there should be a paradigm shift from focusing on economic production to a measurement system that puts emphasis on the well-being of the current and the future generations [1]. Governments in France, Italy, Germany, Spain and China are taking steps to measure the quality of life of their citizens in addition to economic growth [2].

As life expectancy increases and subsequently, the burden of chronic diseases rises, subjective well-being is of growing relevance to the elderly, since subjective well-being is potentially a protective factor for health, reducing the risk of chrosnic physical illness and promoting longevity [3]. Steptoe and colleagues suggested that there are three main aspects of subjective well-being—evaluative, hedonic and eudemonic well-being [4]. Evaluative well-being refers to how satisfied people feel with their lives. Hedonic well-being refers to everyday affects and feelings, such as happiness, sadness, anger and stress. Eudemonic well-being evaluates judgements about one’s feeling about the meaning of his/her life [4]. Subjective well-being is affected by many factors. In addition to one’s status of health playing a pivotal role in affecting subjective well-being [5], material conditions and socioeconomic status also appears to be important, at least for one or all aspects of subjective well-being. In the US, higher income and education level have been shown to be related to better hedonic and evaluative well-being [6]. In Hong Kong, a previous study found a social gradient of self-perceived social status and health-related quality of life [7].

However, in the elderly population, the relationship between household income and evaluative well-being appeared to be heterogeneous across settings [8]. It is also not clear whether income at neighbourhood or district level is related to subjective well-being in the elderly population. One cross-sectional study in Canada showed that neighbourhood income is related to subjective well-being, but not at provincial level [9]. However, little research has examined this in a recently developed setting. 

Hong Kong has one of the highest annual gross domestic product (GDP) per capita in the world, but income inequality has increased following the rapid economic development since the 1960s. The Gini coefficient, a measure of income inequality, has increased from 0.43 in the 1970s to 0.54 in the early 2000s [10]. The median monthly household income varies by District Council district (range from HKD$22,000 in Kwun Tong district to HKD$42,000 in Wan Chai district) [11]. The difference between the monthly median income of males and females in Hong Kong has also been increasing to HKD$6500 in 2018 from HKD$6000 in 2016, and poverty is more prevalent in females than males (in 2017, female 15.3% vs. male 14.6%, after government redistribution interventions) [12]. Moreover, despite the implementation of governmental social security schemes, such as the comprehensive social security assistance (CSSA) and old age living allowance (OALA), the poverty rate (individuals earning below 50% of the median income) of the elderly still stand at 30.9% in 2018 [13]. Locally, the relationships between the macro socioeconomic environment and people’s well-being have seldom been investigated, especially for the elderly population. One previous local study reported that income inequality at neighbourhood level was not related to self-reported health [14]. 

### Aims and Hypothesis

As such, using a large elderly cohort in Hong Kong, we examined the associations of income, education and income inequality at different levels and subjective well-being. We hypothesised that a social gradient of subjective well-being is present using the three measures of socioeconomic status in this sample of elderly. 

## 2. Materials and Methods 

### 2.1. Participants and Procedure

The current cross-sectional study is part of the baseline well-being assessment of the Jockey Club Community eHealth Care project, a project initially aimed to assess the effectiveness of tele-care programme promoting self-management and preventive interventions of chronic diseases in community-dwelling elderly. Specifically, the programme comprised (1) active follow-up of the elderly by a community nurse team by monitoring the data shared to online cloud servers; (2) regular health assessments and (3) regular calls and outreach visits by a multi-disciplinary team consisting of nurses, health workers and social workers. All community-dwelling elderly aged 60 or above were eligible to participate. Elderly participants were recruited from 80 community centres across 18 districts in Hong Kong from 2016 to 2018. Each elderly participant was given a tablet computer to complete a survey in small groups of 6 to 8. Designated staff members of the respective community centres were trained to guide the elderly participants to finish the survey. This Jockey Club Community eHealth Care project was detailed elsewhere [15,16,17]. The study was approved by the Survey and Behavioral Ethics Committee of the Chinese University of Hong Kong (No. 126-16). The study was performed in accordance with the Declaration of Helsinki. Informed consent was obtained from all participants.

There were 7552 elderly participants (median age 76 years, interquartile range (70 to 82), 79% female) in the current study, who nested within 58 TPUs which the community centres that the elderly attended belong to, which, in turn, nested within the 18 district council districts (Table 1).

### 2.2. Exposure

The exposures of this study were income, education and neighbourhood income inequality. Individual disposable income was assessed from the baseline questionnaire by asking the participants “do you feel that you have adequate money these days?”. The possible responses were “very adequate/adequate/just right/inadequate/very inadequate”. We also scaled the income measure in order to derive a measure of the relative index of inequality (RII) [18,19]. The RII compares the risk of low subjective well-being between elderly of lowest and highest socioeconomic status by ranking the income groups from the lowest to the highest and allocating a score (ranging from 0 to 1) that equals the midpoint of the category’s range in the cumulative distribution. For instance, if 24% of the elderly were in the lowest income category, they would be allocated a score of 0.12, and if the next group of elderly constituted 42%, they would be allocated a score of 0.45 (0.24 + 0.42/2) etc. We used this score as a continuous exposure variable in our regression model. The exponentiated coefficient gives a relative risk (RR), comparing the elderly with the possible highest disposable income with the lowest [18]. 

**Figure 1:**
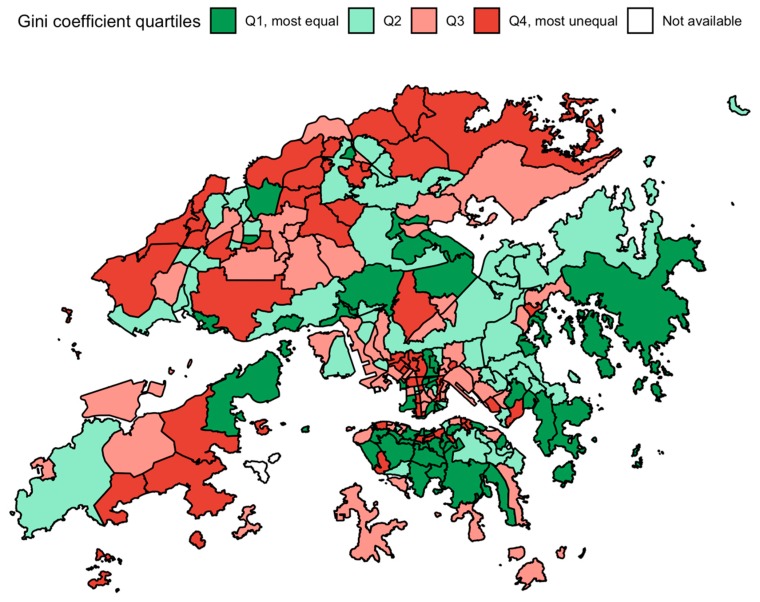
Gini coefficients by tertiary planning units in 2011 in Hong Kong.

We obtained the median monthly income data at the Tertiary Planning Unit (TPU) and district council level from the 2011 population Census in Hong Kong, where the whole territory was divided into 291 TPUs (Figure 1) for town planning purposes and 18 district council districts for administrative purposes. According to the standard methods by the Census and Statistics Department of the government of the Hong Kong Special Administrative Region of China, the 291 TPUs were compressed into 210 TPUs due to small cell size in some of the less populated areas. 

Individual education level was assessed from the baseline questionnaire. Education level was also transformed into RII to assess the risk of low subjective well-being across the top and bottom of the education gradient. Education level at the TPU and the district council district level was obtained as the percentage of the population aged over 15 years with no formal or pre-primary schooling. 

Income inequality at the TPU and district level was computed as Gini coefficients based data on household income, with a range from 0 (completely equal income distribution) to 1 (all income was earned by one person) [20]. We aligned with previous studies by classifying the Gini coefficients into quartiles in order to avoid assuming linearity [14], with quartile 1 being the most equal and 4 being the least equal. We also consider Gini coefficients as a continuous variable in the analysis.

### 2.3. Outcome

The outcome of this study is low subjective well-being. The three different aspects of subjective well-being—evaluative, hedonic and eudemonic—were assessed by the following questions: evaluative well-being: “are you satisfied with your life? (yes/no)”; hedonic well-being: “are you happy with your life? (a Likert scale from 0 to 10, with 0 being the most unhappy and 10 being happiest)”; and eudemonic well-being: “do you find purpose or meaning in your life? (yes/no)”. The participant was classified as having low subjective well-being when he/she answered “no” for evaluative or eudemonic well-being or scored 2 or below for hedonic well-being. 

### 2.4. Statistical Analysis

The data of the current study are of a multilevel structure comprising the elderly participants (level 1), who nested within the TPUs which the community centres belong to (level 2), and which, in turn, nested within the 18 district council districts (level 3). We therefore used multivariable multilevel logistic regression models with random intercept to assess the associations between income, education level, neighbourhood income inequality and low subjective well-being and its individual subdomains of evaluative (not satisfied with life), hedonic (unhappy with life) and eudemonic well-being (no meaning in life). The multilevel model had the following general structure:

$$Logit\left(p_{ijk}\right)=\beta x_{ijk}+\gamma y_{jk}+\eta z_{k}+\omega_{jk}+\varphi_{k}$$

where the probability of having low subjective well-being was predicted by individual levels covariates $x_{ijk}$, TPU level covariates $y_{jk}$ and District level covariates $z_{k}$, in which i=1…I individuals, j=1…J TPUs, k=1…K District council districts and β, γ and η are the regression coefficients for the covariates at the three respective levels and $\omega \sim N\left(0,\sigma_{TPU}^{2}\right)$ and $\varphi \sim N\left(0,\sigma_{district}^{2}\right)$ are the error terms for TPU and district levels. The structure of a random intercept was built based on the knowledge of the hierarchical structure of the data and whether a random slope was needed was assessed by Akaike information criterion (AIC).

Covariates were chosen on the basis of being common causal precedents of both the exposure and the outcome, or potentially on the confounding pathways [21]. We adjusted for age and sex for all models. For the model assessing the association of income and low subjective well-being, we additionally adjusted for education level, whilst for the model assessing the association of income inequality and low subjective well-being, we additionally adjusted for disposable income and education level. Odds ratios (ORs) and their corresponding 95% confidence intervals (95%CI) were reported. The analyses were then repeated to obtains the estimates for the each of the three components of subjective well-being. Whether the associations varied by age and sex was assessed by the significance of the interaction term.

To check whether the findings were robust to unadjusted confounding, we conducted a sensitivity analysis by applying the bounding factor

$$\frac{OR_{EU}\times OR_{UD}}{OR_{EU}+OR_{UD}-1}$$

developed by Ding and Vanderweele to our regression estimates [22], in which OR_EU_ represents to the association of the unmeasured confounder and the exposure and OR_UD_ represents the association of unmeasured confounder and the outcome. All analyses were undertaken using R (version 3.5.1).

## 3. Results

### 3.1. Baseline Characteristics

Among the participants, 15.3% (95%CI 14.5 to 16.1) had low subjective well-being. Splitting low subjective well-being into the three components, 2.4% (95%CI 2.0 to 2.8) of the participants reported being unhappy, 8.4% (95% CI 7.8 to 9.1) of them were not satisfied with their lives and 10.2% (95%CI 9.5 to 10.9) did not find meaning or purpose in their lives. There were 1.5% (95%CI 1.2 to 1.8) of them who felt that they have a very adequate disposable income, whereas 4.2% (95%CI 3.8 to 4.7) of them felt that their income is very inadequate. There were 4.6% (95%CI 4.2 to 5.1) of the participants with a tertiary education or above, whereas around a quarter of them had received no formal schooling (25.9%, 24.9 to 26.9). Around 80% of the participants were females. 

### 3.2. Multilevel Analysis

At individual level, lower disposable income was associated with increased risk of subjective well-being (Table 2) (RII = 6.55; 95%CI 4.98 to 8.60). For the three components of subjective well-being, a lower disposable income was strongly associated with being unhappy (RII = 13.69; 95%CI 7.42 to 25.28) and not satisfied with life (RII = 13.34; 95%CI 9.44 to 18.87). A low disposable income was also associated with not having found meaning or purpose in life (RII = 4.02; 95%CI 2.93 to 5.53). On the district level, a lower income at the TPU level was associated with lower subjective well-being (OR = 0.82, 95%CI 0.70 to 0.98) and not having found meaning or purpose in life (OR = 0.74; 95%CI 0.59 to 0.93). The income at district council district level did not seem to associate with subjective well-being and its components. The above associations did not vary by age and sex. 

A lower education level was associated with higher risk of low subjective well-being (RII = 1.60; 95%CI 1.22 to 2.09) and not having found meaning and purpose in life (RII = 1.39; 95%CI 1.01 to 1.92) (Table 3). However, the associations at individual level varied by age but not by sex and stratified results were presented in Table A1 (in Appendix A) (p for interaction with age: low subjective well-being = 0.02, being unhappy = 0.004, not satisfied with life = 0.02, no purpose or meaning in life = 0.10). The associations of lower education and higher risk of low subjective well-being and its components was generally stronger with increasing age. At TPU level, a higher proportion of population with pre-primary education/no formal schooling in the TPU was associated with higher risk of low subjective well-being (OR = 1.11; 95%CI 1.04 to 1.18), being not satisfied with life (OR = 1.07; 95%CI 1.02 to 1.13) and not having found meaning or purpose in life (OR = 1.14; 95%CI 1.05 to 1.24). Conversely, at district level, a higher proportion of the population with pre-primary/no formal schooling was associated with a lower risk of low subjective well-being (OR = 0.85; 95%CI 0.73 to 0.98), not satisfied with life (OR = 0.86; 95%CI 0.76 to 0.97) and not having found meaning or purpose in life (OR = 0.79; 95%CI 0.66 to 0.96). 

**Figure A1:**
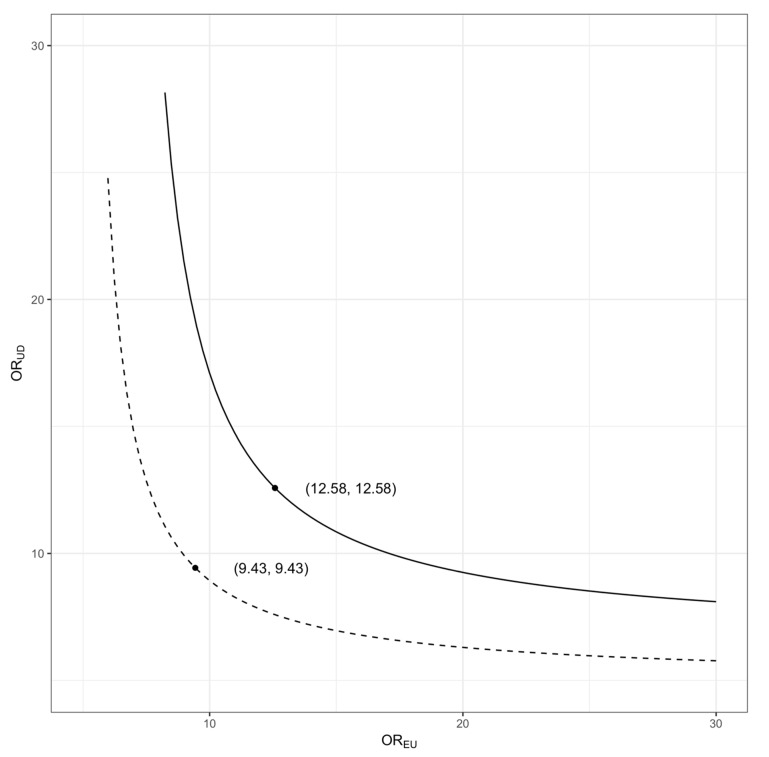
Sensitivity analysis for the association of disposable income and low subjective well-being.

**Figure A2:**
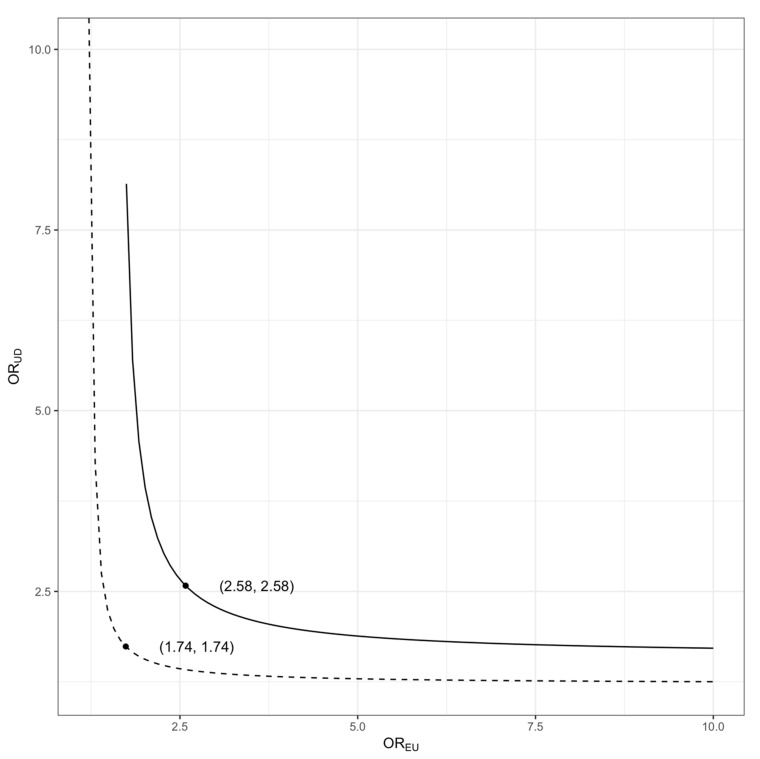
Sensitivity analysis for the association of education and low subjective well-being.

Income inequality was not associated with subjective well-being and its components at TPU and district levels (Table 4). Our sensitivity analysis shows that the association between disposable income and low subjective well-being is robust to strong unmeasured confounding in order to bias the observed association to the null (OR required ≥ 9.43) (Figure A1). However, the observed association of education and low subjective well-being might be susceptible to unmeasured confounding of moderate strength (OR required ≥ 1.74) (Figure A2). 

## 4. Discussion

In a recently developed non-Western setting, the present cross-sectional study found that elderly with more adequate disposable income were more likely to have better subjective well-being, whereas those with higher education also appeared to be less likely to suffer from poorer subjective well-being, but this link was generally stronger among those who are older. At TPU level, higher income and lower proportion of population with low education is related to lower risk of subjective well-being. Conversely, a higher proportion of the population with low education level at district level is related to better subjective well-being. Income inequality at neighbourhood level did not seem to relate to subjective well-being. 

### 4.1. Comparison with Previous Studies

The finding that disposable income was associated with subjective well-being and its components is consistent with the literature. A previous small cross-sectional study (n=450) in Hong Kong found that self-reported economic condition was positively related to self-rated health and mental health status in the elderly [23]. The Gallup-Healthways Well-Being Index study surveyed over 450,000 US citizens in 2008 to 2009 and found that household income was related to both hedonic and eudemonic well-being [6]. The current study adds to the previous literature that this is the first study investigating the social gradient of the complex construct of subject well-being and its components in a Chinese elderly population.”

We found that neighbourhood income inequality were not related to subjective well-being and it is largely consistent with a previous local study using data from the population-representative Thematic Household Survey that reported income inequality at the district and TPU level was not related to self-reported health [14]. As such, our findings do not seem to corroborate with the relative income hypothesis, which suggested that income inequality within a neighbourhood rather than absolute level of individual socioeconomic deprivation that does the damage to health [24]. Hong Kong is very densely populated, such that more well-off private housing may be situated in the immediate vicinity of poorer public housing estates. It also has good connectivity by affordable means of transportation. Thus, these might have contributed to our null findings given the relative homogeneity across neighbourhoods. It also might nonetheless hinge on the level of population aggregation. In this study, neighbourhood socioeconomic indicators appeared to show qualitatively different associations with subjective well-being and its components at different hierarchical level of the data. Recently, population size has been reported as a factor that modifies the relationship between relative income deprivation and perceived health [25]. In general, the larger the population unit being studied, the greater the degree of unobserved heterogeneity within neighbourhoods [26]. In this regard, it could become harder to interpret what the socioeconomic indicator represents at these higher levels. However, we could possibly rule out that area median household income or percentage of population with no formal schooling reflects income inequality given our results (Table 4). One possible interpretation here is that higher neighbourhood income and better education is generally related to better subjective well-being in the elderly, at least at TPU level. Nonetheless, in order to be more certain about the direction of the relationship or be able to draw causal conclusions, future studies could employ fixed effect models to longitudinal data to control for time-invariant unmeasured heterogeneity over time [27].

The finding that higher education was related to better subjective well-being is consistent with the well-established literature across numerous settings [28,29]. However, the reason for larger educational gradient of subjective well-being and, unhappiness in particular, for older compared to younger participants is not immediately clear. Such an age pattern of inequality in health outcome was similarly reported in a previous study using data from 11 European countries and found that it varies across countries, i.e., some reported smaller inequality of mortality in some countries while some reported the opposite as age increases [30]. This pattern could possibly arise from age difference in the associations of some mediators and subjective well-being. One of the possibilities is that lower education might have led to loneliness in the elderly, which in turn could affect subjective well-being, and advancing age could possibly modify the latter relationship [31]. Another possibility is that education tended to be more accessible with time, so those who are older might show a steeper health inequality by education level than the younger elderly.

### 4.2. Strengths and Limitations

The current study made use of three independent indicators of socioeconomic status to examine the different aspects of the complex construct of subjective well-being, whereas traditional surveys of subjective well-being mainly focused on eudemonic well-being [6]. We used information concerning subjective assessment of disposable income of the elderly participants, which is seldom examined in the literature [23]. From a cultural point of view, self-reported disposable income might go beyond objective monetary terms but to reflect family support and sense of security [32]. We also used modern techniques of sensitivity analysis to assess whether the results obtained were robust to unmeasured confounding.

However, several potential caveats should also be borne in mind when interpreting our findings. First, since this is a cross-sectional study, it is inevitable that such design is not able to establish causality between exposure and outcome. We could not therefore rule out the possibility that previous subjective well-being could have affected how the elderly rated his or her financial status. Repeated measurement of financial status and subjective well-being will be possible as there are ongoing cohort follow-ups but the current study could only capitalise on the baseline data. Second, subjective well-being and its components were not measured by validated psychometric tools in this study. However, we are aware that the measurement and definition of subjective well-being is an extensive research area in itself [33]. Thus, a definite or validated way or measuring this construct has not been reached in the academic discourse. Conversely, we deem that the relatively straightforward derivation of the measure of subjective well-being should enable clinicians or researchers to implement it to a wider elderly population and observe trend over time if possible. Third, we could not assess how closely correlated are self-reported disposable income and the actual financial conditions of the elderly participants given we did not have the data for the latter. However, previous studies have suggested that subjective experience of financial conditions might be more closely related to health outcomes than the actual level of income or wealth, especially for the elderly [23], given income might be a less sensitive measure of socio-economic position in later life after retirement [34]. We therefore believe that subjective assessment of financial condition should suffice to measure adequacy at meeting personal and financial needs. Fourth, we are aware that this sample has a relatively substantial imbalance in sex ratio. However, we included sex in all of our models and simulation studies have shown that conventional regression adjustment suffice to correct the covariate imbalance [35]. Finally, we could not rule out the possibility that both subjective well-being and self-perceived disposable income are affected by some common unmeasured causal antecedents. For instance, individuals who reported better well-being might also rate their financial conditions more favourably, perhaps they have better mental health to start with (confounding). However, another individual level measure of socioeconomic position, i.e., education, showed similar pattern of association with subjective well-being. The sensitivity analysis also suggests that the current analysis is robust to unmeasured confounding. 

### 4.3. Policy Implications

Following medical advancement, people are expected to live increasingly longer lives. Health-care policies thus should be concerned not only on physical but also mental well-being of the elderly population, as well as the more upstream supporting methods that improve subjective well-being of the elderly. For instance, adequate financial resources are important for healthy diet and housing of the elderly and hence affecting their physical and mental health [23]. Hence, relieving the elderly from financial strain, which is a day-to-day stressor that affects the psychological health and well-being, could be of substantial policy benefit [36]. In addition, future studies should explore how strong or whether a threshold effect is present for the association between disposable income and objective measurement of wealth in elderly in Hong Kong, and whether this association would be different between neighbourhoods. Such knowledge would inform welfare policies regarding the establishment of a safety net. 

## 5. Conclusions

Elderly with inadequate disposable income and lower education level are more likely to suffer from low subjective well-being. At neighbourhood level, income inequality did not seem to relate to subjective well-being. However, the relationships of neighbourhood income and education level and individual’s subjective well-being were not clear. A social gradient of subjective well-being is evident among elderly in Hong Kong. The public health policy agenda should reprioritise to shed more light on subjective well-being of the elderly given the ageing population, especially by relieving their financial burden. 

## Figures and Tables

**Table 1:** Baseline characteristics of the cohort participants of the Jockey Club Community eHealth Care project.

n (%) for Categorical Variables or Median [IQR] for Continuous Variables	n = 7552
Low subjective well-being	1156 (15.3)
Unhappy with life (0-2 on Likert scale)	182 (2.4)
Not satisfied with life	635 (8.4)
No meaning in life	771 (10.2)
Disposable income	
Very adequate	110 (1.5)
Adequate	958 (12.7)
Just right	4887 (64.7)
Inadequate	1279 (16.9)
Very inadequate	318 (4.2)
Level of education	
Tertiary or above	348 (4.6)
Secondary education	1867 (24.7)
Primary education	3381 (44.8)
No formal schooling	1956 (25.9)
Age	76.00 [70.00, 82.00]
Female	5976 (79.1)
Chronic conditions	
Hypertension	4666 (61.8)
Diabetes	2013 (26.7)
Hypercholesterolaemia	2223 (29.4)
Heart diseases	992 (13.1)
Stroke	354 (4.7)
Chronic obstructive pulmonary disease	191 (2.5)
Renal disease	99 (1.3)

Abbreviation: IQR: interquartile range.

**Table 2:** Multilevel associations of income and components of and overall subjective well-being.

	Odds Ratio (95%CI)			
	Low SWB	Unhappy with LIFE	Not Satisfied with Life	No Meaning in Life
*Individual level*				
Very adequate	Ref.	Ref.	Ref.	Ref.
Adequate	1.05 (0.51 to 2.17)	0.40 (0.08 to 1.97)	1.15 (0.34 to 3.82)	1.48 (0.58 to 3.79)
Just right	1.74 (0.87 to 3.49)	0.96 (0.23 to 3.96)	2.61 (0.82 to 8.26)	2.06 (0.83 to 5.11)
Inadequate	4.08 (2.02 to 8.23)	2.38 (0.57 to 9.93)	7.70 (2.42 to 24.50)	4.04 (1.62 to 10.10)
Very inadequate	5.08 (2.44 to 10.59)	5.77 (1.35 to 24.60)	10.91 (3.36 to 35.47)	4.22 (1.63 to 10.96)
RII	6.55 (4.98 to 8.60)	13.69 (7.42 to 25.28)	13.34 (9.44 to 18.87)	4.02 (2.93 to 5.53)
*Neighbourhood level*				
Income at TPU level	0.82 (0.70 to 0.98)	0.77 (0.56 to 1.05)	0.87 (0.75 to 1.02)	0.74 (0.59 to 0.93)
Income at district level	1.13 (0.94 to 1.37)	1.01 (0.81 to 1.26)	1.06 (0.92 to 1.23)	1.18 (0.94 to 1.49)

Models adjusted for age, sex and education level. Neighbourhood level estimates are interpreted as the change in odds per standard deviation (SD) increase of neighbourhood income (SD at TPU level = $11060.53; SD at district level = $5660.08). RII estimates are interpreted as the change in odds from the bottom to the top of the gradient of disposable income. Abbreviations: CI = confidence interval; RII = relative index of inequality; SWB = subjective well-being; TPU = tertiary planning unit.

**Table 3:** Multilevel associations of education and components of and overall subjective well-being.

	Odds Ratio (95%CI)			
	Low SWB	Unhappy with Life	Not Satisfied with Life	No Meaning in Life
*Individual level*				
Tertiary education	Ref.	Ref.	Ref.	Ref.
Secondary education	0.99 (0.71 to 1.39)	0.81 (0.40 to 1.64)	0.79 (0.53 to 1.17)	0.98 (0.67 to 1.44)
Primary education	1.08 (0.78 to 1.49)	0.70 (0.35 to 1.40)	0.80 (0.55 to 1.17)	0.99 (0.68 to 1.44)
No formal schooling	1.41 (1.00 to 1.98)	1.33 (0.65 to 2.72)	1.08 (0.72 to 1.62)	1.27 (0.86 to 1.89)
RII	1.60 (1.22 to 2.09)	1.83 (0.98 to 3.41)	1.40 (0.99 to 1.97)	1.39 (1.01 to 1.92)
*Neighbourhood level*				
No schooling/pre-primary % at TPU level	1.11 (1.04 to 1.18)	1.06 (0.98 to 1.14)	1.07 (1.02 to 1.13)	1.14 (1.05 to 1.24)
No schooling/pre-primary % at district level	0.85 (0.73 to 0.98)	0.97 (0.81 to 1.17)	0.86 (0.76 to 0.97)	0.79 (0.66 to 0.96)

Models adjusted for age and sex. Neighbourhood level estimates are interpreted as the change in odds per percentage point increase of population with no schooling/pre-primary education in the area. RII estimates are interpreted as the change in odds from the bottom to the top of the gradient of disposable income. Abbreviations: CI = confidence interval; RII = relative index of inequality; SWB = subjective well-being; TPU = tertiary planning unit.

**Table 4:** Multilevel associations of neighbourhood income inequality and components of and overall subjective well-being.

	Odds Ratio (95%CI)			
	Low SWB	Unhappy with Life	Not Satisfied with Life	No Meaning in Life
*Neighbourhood level*				
**TPUs**				
Gini coefficient				
1st quarter (most equal)	Ref.	Ref.	Ref.	Ref.
2nd quarter	1.57 (0.81 to 3.04)	1.57 (0.71 to 3.45)	1.45 (0.85 to 2.46)	1.98 (0.87 to 4.53)
3rd quarter	1.40 (0.73 to 2.69)	1.21 (0.55 to 2.67)	1.26 (0.75 to 2.13)	1.71 (0.76 to 3.88)
4th quarter (most unequal)	1.48 (0.68 to 3.26)	1.44 (0.58 to 3.60)	1.39 (0.74 to 2.59)	1.92 (0.72 to 5.10)
Gini as continuous	1.02 (0.97 to 1.07)	1.01 (0.95 to 1.07)	1.02 (0.98 to 1.06)	1.04 (0.98 to 1.11)
**Districts**				
Gini coefficient				
1st quarter (most equal)	Ref.	Ref.	Ref.	Ref.
2nd quarter	0.90 (0.58 to 1.41)	0.60 (0.37 to 1.00)	1.07 (0.76 to 1.51)	0.78 (0.45 to 1.34)
3rd quarter	0.87 (0.54 to 1.38)	0.75 (0.47 to 1.20)	0.99 (0.69 to 1.40)	0.82 (0.46 to 1.44)
4th quarter (most unequal)	0.93 (0.57 to 1.49)	0.73 (0.45 to 1.18)	1.10 (0.76 to 1.57)	0.83 (0.47 to 1.50)
Gini as continuous	0.98 (0.86 to 1.11)	0.93 (0.80 to 1.08)	1.01 (0.91 to 1.11)	0.95 (0.81 to 1.11)

Models adjusted for age, sex, disposable income and education level. Estimates for Gini as continuous are interpreted as the change in odds per 0.01-point increase in neighbourhood Gini coefficients. Cut-offs for TPU Gini coefficients are 1st: (0.074, 0.389), 2nd: (0.379, 0.423), 3rd: (0.423, 0.444), 4th: (0.444, 0.546). Cut-offs for district Gini coefficients are: 1st: (0.409, 0.425), 2nd: (0.425, 0.429), 3rd: (0.429, 0.440), 4th: (0.440, 0.463). Abbreviations: CI = confidence interval; SWB = subjective well-being; TPU = tertiary planning unit.

## Appendix A

**Table A1:** Multilevel associations of education and components of and overall subjective well-being, stratified by age groups.

	RII (95%CI)			
Age Groups	Low SWB	Unhappy with Life	Not Satisfied with Life	No Meaning in Life
*Individual level*				
Young-old (60 to 69)	1.26 (0.70 to 2.27)	0.69 (0.20 to 2.33)	1.12 (0.54 to 2.30)	1.23 (0.62 to 2.47)
Old-old (70 to 79)	1.83 (1.21 to 2.77)	2.12 (0.75 to 5.96)	1.57 (0.92 to 2.66)	1.66 (1.02 to 2.71)
Oldest-old (80+)	1.70 (1.07 to 2.69)	3.14 (1.02 to 9.64)	1.43 (0.80 to 2.57)	1.40 (0.81 to 2.43)

Multilevel models adjusted for age and sex, with random intercepts by TPU and district council districts incorporated. Abbreviations: RII = relative index of inequality; SWB = subjective well-being.

The x-axis represents the association of the unmeasured confounder (U) and the exposure (disposable income, E), whilst the y-axis represents the association of the unmeasured confounder (U) and the outcome (low subjective well-being, D). The area above the solid line is the joint values of the E-U and U-D associations that would be required to explain away the observed E-D association (RII = 6.55). The highlighted point on the line represents the strength of either of the E-U or U-D associations has to be bigger than that value in order to explain away the observed E-D association. The dashed line represents that of the lower bound of the 95% confidence interval (4.98). Overall, it means that in the case if either of the E-U or U-D associations has an odds ratio ≥ 9.43, the observed E-D association would be explained away to the null.

The x-axis represents the association of the unmeasured confounder (U) and the exposure (education, E), whilst the y-axis represents the association of the unmeasured confounder (U) and the outcome (low subjective well-being, D). The area above the solid line is the joint values of the E-U and U-D associations that would be required to explain away the observed E-D association (RII = 1.60). The highlighted point on the line represents the strength of either of the E-U or U-D associations has to be bigger than that value in order to explain away the observed E-D association. The dashed line represents that of the lower bound of the 95% confidence interval (1.22). Overall, it means that in the case if either of the E-U or U-D associations has an odds ratio ≥ 1.74, the observed E-D association would be explained away to the null.
